# Red Blood Cells in the Cerebrospinal Fluid Compartment After Subarachnoid Haemorrhage: Significance and Emerging Therapeutic Strategies

**DOI:** 10.1007/s12975-024-01238-9

**Published:** 2024-02-29

**Authors:** Soham Bandyopadhyay, Nina Schwendinger, Behnam Rezai Jahromi, Shivanand P. Lad, Spiros Blackburn, Stefan Wolf, Diederik Bulters, Ian Galea, Michael Hugelshofer

**Affiliations:** 1 Clinical Neurosciences, Clinical & Experimental Sciences, Faculty of Medicine, University of Southampton, Southampton, Hampshire UK; 2 Wessex Neurological Centre, University Hospital Southampton NHS Foundation Trust, Southampton, UK; 3 Department of Neurosurgery, Clinical Neuroscience Center, Universitätsspital and University of Zurich, Zurich, Switzerland; 4 Department of Neurosurgery, Helsinki University Hospital and University of Helsinki, Helsinki, Finland; 5 Department of Neurosurgery, Duke University Medical Center, Durham, NC USA; 6 Department of Neurosurgery, University of Texas Houston Health Science Center, Houston, TX USA; 7 Department of Neurosurgery, Charité–Universitätsmedizin Berlin, Corporate Member of Freie Universität Berlin, Humboldt-Universität zu Berlin, and Berlin Institute of Health, Berlin, Germany; 8 Department of Neurosurgery, University Hospital Mannheim, Medical Faculty Mannheim, University of Heidelberg, Mannheim, Germany

**Keywords:** Subarachnoid haemorrhage, Blood volume, Haemoglobin, Lumbar drain, Neurapheresis, Haptoglobin

## Abstract

Subarachnoid haemorrhage (SAH) is a subtype of stroke that predominantly impacts younger individuals. It is associated with high mortality rates and can cause long-term disabilities. This review examines the contribution of the initial blood load and the dynamics of clot clearance to the pathophysiology of SAH and the risk of adverse outcomes. These outcomes include hydrocephalus and delayed cerebral ischaemia (DCI), with a particular focus on the impact of blood located in the cisternal spaces, as opposed to ventricular blood, in the development of DCI. The literature described underscores the prognostic value of haematoma characteristics, such as volume, density, and anatomical location. The limitations of traditional radiographic grading systems are discussed, compared with the more accurate volumetric quantification techniques for predicting patient prognosis. Further, the significance of red blood cells (RBCs) and their breakdown products in secondary brain injury after SAH is explored. The review presents novel interventions designed to accelerate clot clearance or mitigate the effects of toxic byproducts released from erythrolysis in the cerebrospinal fluid following SAH. In conclusion, this review offers deeper insights into the complex dynamics of SAH and discusses the potential pathways available for advancing its management.

## Introduction

Subarachnoid haemorrhage (SAH) is a life-threatening and debilitating type of stroke, characterised by bleeding into the subarachnoid space around the brain [1]. Spontaneous SAH accounts for approximately 5% of all strokes [2] with the most common cause of a spontaneous SAH being the rupture of a cerebral artery aneurysm at an incidence of 6.1 per 100,000 person-years [3]. Although SAH is less prevalent than other types of strokes, its impact is disproportionally severe. It predominantly affects younger, working-age individuals [4]. Approximately one third of SAH patients die [5–7], and survivors face significant long-term physical, cognitive, and psychosocial challenges [8], often resulting in unemployment and reduced quality of life [9].

Given the societal burden caused by SAH, there has been an urgent need to characterise the pathophysiology of SAH and to identify novel treatments to improve outcomes. The initial bleeding event introduces large numbers of red blood cells (RBCs) into the cerebrospinal fluid (CSF) compartment [10]. These RBCs may be partially cleared by erythrophagocytosis [11, 12]. However, the magnitude of haemorrhage in SAH far exceeds the capacity of phagocytic clearance pathways. Consequently, extravasated RBCs start to lyse within the CSF and release toxic haemoglobin (CSF-Hb). CSF-Hb is subsequently broken down into its constituent haem groups and globin proteins [13]. CSF-Hb—which can permeate cerebral arterial walls and cortical tissue—has been increasingly recognised as a root cause of secondary brain injury after SAH (SAH-SBI) [14, 15].

This review aims to provide a comprehensive synthesis of current research focusing on the relevance of the initial blood load and clot clearance in SAH, and how these factors correlate with SAH-SBI and patient outcomes. It will critically assess the methodologies used for clot quantification and discuss potential differences in pathophysiology depending on the anatomical location of the haematoma. Additionally, the review will discuss emerging therapeutic strategies targeting RBCs or RBC-derived toxins and describe future research directions.

The terminology on outcome measures used in the original publications has been retained in this review to avoid a false interpretation of the original results. Therefore, although outdated, the term symptomatic vasospasm will be encountered in this review.

## Initial Blood Load

Numerous studies have investigated if the prognosis of SAH patients is linked to the volume, density, and/or the anatomical location of the haematoma.

Early research in this area was aimed at developing qualitative CT-based radiographic scales that could use blood volume and blood location for predicting the likelihood of delayed cerebral ischaemia (DCI) following SAH. A recent study found that of the radiological grading systems created, the Hijdra sum score was the only scale with good inter-observer reliability [16]. With an area under the curve (AUC) for functional outcome at 6 months of 0.76, the Hijdra sum score outperformed other scales like the original Fisher, which had an AUC of 0.67 [16]. This reflects incremental improvements in the detail of the grading systems, but even the most detailed are qualitative approximations that do not provide the same accuracy as volumetrically quantifying the blood load.

These grading systems also do not consider the density of the haematoma. The Hounsfield unit (HU) value in a CT scan reflects haematoma density and likely correlates with RBC content of the clot [17]. It was found that the average HU of blood clots located in the cistern was significantly associated with symptomatic vasospasm [18]. This indicates a potential prognostic value of considering clot density.

The advent of volumetric quantification techniques has provided a step improvement over qualitative scales for quantifying blood volume on CT scans. This approach demonstrates greater discriminatory power in determining prognosis compared to a traditional radiographic scale [19]. Although this technique was initially more cumbersome than using radiographic scales as it required manual segmentation of the scan, automation has drastically reduced the time by at least two thirds without compromising on the accuracy of the volumetric readings [20]. A recent study indicates that a model using automated volumetric quantification of the haemorrhage offers a 25% greater predictive power of long-term prognosis compared to the same model employing a radiological grading system, and has an AUC for functional outcome of 0.89 [21]. The same study showed that considering the cisternal, ventricular, and parenchymal volumes instead of total blood volume in the model separately did not improve the predictive value of the model [21], suggesting that blood in each of these compartments is deleterious.

This is consistent with a large amount of literature regarding the role of intraventricular haemorrhage (IVH), which has been consistently associated with worse outcomes post-SAH. The largest of these studies pooled data from four randomised controlled trials in which the presence or absence of IVH was associated with poor neurological outcomes at 3 months, even after controlling for confounding factors [22]. In addition, IVH was significantly associated with developing hydrocephalus in this study; however, this was not controlled for as a confounding factor when looking at the association between IVH and long-term outcomes. It may be the case that there are other factors causing poor CSF clearance that are associated with poor outcomes. Another study used a proxy marker of IVH volume called the IVH score, which includes hydrocephalus to determine the association between the volume of IVH and poor outcomes. They found that each millilitre increase in IVH—calculated from the IVH score—increased the odds of a poor outcome by 1.11 (even after adjusting for WFNS) [23], but was not associated with DCI.

In terms of DCI, a study conducted just over a decade ago found that the total blood volume in the ventricles and the cisterns was significantly associated with DCI in a dose-related manner, and this remained the case after adjusting for admission Hunt-Hess grade [24]. Patients in the highest total blood volume quartile also had a higher risk of death and poor modified Rankin Scale (mRS) outcome at 3 months compared to those with lower total blood volumes. Furthermore, subdividing the blood clot volume into its ventricular and cisternal components revealed that patients with DCI had a higher amount of blood in both the ventricles and the cisterns. However, this study did not report on the relationship between IVH and DCI after adjusting for the effect of cisternal blood volume. A more recent study quantified total blood volume in SAH patient using an automatic haemorrhage-segmentation algorithm, where blood was manually classified as cisternal, intraventricular, intraparenchymal, or subdural [25]. They found that a per millilitre increase in total blood volume (OR = 1.02; 95% CI, 1.01–1.03) or cisternal blood volume (OR = 1.02; 95% CI, 1.01–1.04) significantly increased the odds of DCI, but a per millilitre increase in IVH was not significantly associated with DCI. Therefore, the primary driver of DCI may be related to cisternal blood volume, and the damaging mechanism of IVH may not be through DCI but alternative neuronal injury or inflammatory pathways. A recent study correlating subarachnoid blood volume with DCI found that subarachnoid blood volume in the interhemispheric fissure correlated significantly with DCI in the anterior cerebral artery territory, whilst subarachnoid blood volume in the Sylvian fissure correlated significantly with DCI in the middle cerebral artery (MCA) territory. However, on Bonferroni correction, only subarachnoid blood volume on the cerebral convexity was associated with DCI (in the MCA territory) [26]. The paravascular glymphatic pathway provides a route for subarachnoid blood to enter the cerebral cortex [27], and quantifying the volume of blood that lies in the sulci on the cerebral convexity may improve the prediction of DCI.

## The Relationship Between RBCs and Blood Load

The idea that a higher haematoma volume might be associated with higher CSF RBC counts was first demonstrated by a study showing a positive correlation between CSF RBC counts and radiological grading systems: modified Fisher scale and Hijdra sum score [28]. This suggested that CSF RBC counts—like blood load—could be predictive of various clinical endpoints in SAH.

In this same retrospective study, the authors evaluated the predictive value of initial CSF RBC count on functional outcomes after SAH. CSF samples were obtained from an external ventricular drain on day 2 after SAH. The authors reported an inverse correlation of CSF RBC counts with good outcomes and hospital survival. They used a multivariable binary logistic regression correcting for age and Hunt-Hess grade, but did not correct for hydrocephalus [28]. Another recent study found that elevated RBC counts between days 5 and 18 after SAH were significantly associated with DCI [29]. There is also evidence that CSF RBC counts are predictive of hydrocephalus [30].

## Clot Clearance

The role of RBC-derived toxins as disease-defining modulators in SAH is becoming increasingly evident. In concordance, haematoma clearance could have predictive and therapeutic potential. A faster clearance would result in a lower exposure to RBC degradation products, likely to improve outcome. The following section starts by discussing endogenous clot clearance kinetics before moving on to address therapeutic approaches that could accelerate clot clearance.

### Kinetics of Endogenous Haematoma Clearance

Little is known about endogenous blood clot-clearance kinetics in the CSF space. According to the literature, the clot clearance rate in the ventricles seems to follow first-order kinetics with a percentage rate of clot resolution of about 10.8% daily. While the absolute rate of clot resolution was significantly dependent on the initial clot volume, the percentage rate of clot resolution was independent of the initial clot volume, age, sex, and ventricular drainage [31]. Another study reported a combined clearance rate of intracisternal and intraventricular blood of 18–22% after 1 day, 49–52% after 4 days, and 74–78% after 10 days [32].

### Endogenous Clot Clearance Rate as a Predictor for SBI

Some studies aimed to incorporate the clot clearance rate in their radiographic prediction model for outcomes after SAH. Exploring the association with DCI, recent findings showed distinct results for different anatomical compartments. A study explored the intraventricular and cisternal clot volumes and their respective clearance rates. A multivariate logistic regression correcting for age, GCS at admission, acute hydrocephalus, and the Fisher scale found that a lower cisternal clot clearance rate was significantly associated with a higher risk of DCI. On the other hand, the intraventricular clot clearance rate was not found to be significantly associated with DCI [33]. Supporting a primary role of cisternal SAH, another study concluded that a combination of initial subarachnoid clot volume and the percentage of clot cleared by day significantly predicted the occurrence of vasospasm [34].

Similar results were obtained in a recent study which did not anatomically separate intraventricular and intracisternal blood. The authors retrospectively analysed data from patients after SAH to study the association between clot clearance rate and outcomes. Patients in that cohort had a CT scan at admission and three to four days after SAH. The authors used the Hijdra sum score to assess the intracisternal and intraventricular clot volume. The reduction in blood load was defined as a relative or absolute change between the two-time points. A higher relative Hijdra sum score reduction was significantly associated with a reduced risk for DCI and a higher chance of being home on post-SAH day 30 [32]. One study utilising the combined blood volume of cisternal, and intraventricular blood presented opposing results, with no significant association between the clot clearance rate and DCI [24]. The conflicting results obtained in studies examining combined intracisternal and intraventricular clot clearance rates might be explained by different approaches to assessing the blood volume. One study used a volumetric approach for the combined clot clearance rate; the other utilised the Hijdra sum score. Inspecting these two methods, the intraventricular clot volume is likely to have a predominant influence in the volumetric approach, while the Hijdra method assigns up to 30 points to cisternal blood but only 10 to intraventricular blood volume, thereby putting more weight on the first. Taken together it seems that cisternal, but not ventricular clot clearance rate, is associated with DCI.

## Therapeutic Acceleration of Clot-Clearance

Thrombolytics have been theorised as a therapy to achieve a faster clearance of the blood clot in IVH and SAH.

### Intraventricular Thrombolysis for Intraventricular Haemorrhage

The largest and most prominent trial for thrombolysis in IVH has been the CLEAR III trial [35]. It was a randomised, double-blinded, placebo-controlled, multiregional trial to understand if intraventricular clot removal with alteplase versus saline irrigation improved functional outcomes in patients with spontaneous IVH. Five hundred patients with an IVH and routine external ventricular drain (EVD) placement were randomised to either treatment. Those given alteplase had a smaller volume of IVH at the end of their treatment. At 6 months follow-up, the treatment group had lower mortality but a higher proportion of patients with unfavourable neurological outcome (mRS 5).

Data from the CLEAR III study was also used to examine the effects of intraventricular alteplase treatment on sterile CSF inflammation and leukocyte subsets [36, 37]. The CSF WBC count was significantly higher in the alteplase group; however, CSF leukocyte subsets and numbers did not influence the overall outcomes. Overall, this suggests that thrombolysis accelerated clot lysis and augmented the central inflammatory response, but did not alter long-term functional outcomes.

### Intraventricular Thrombolysis for SAH

**Fig. 1:**
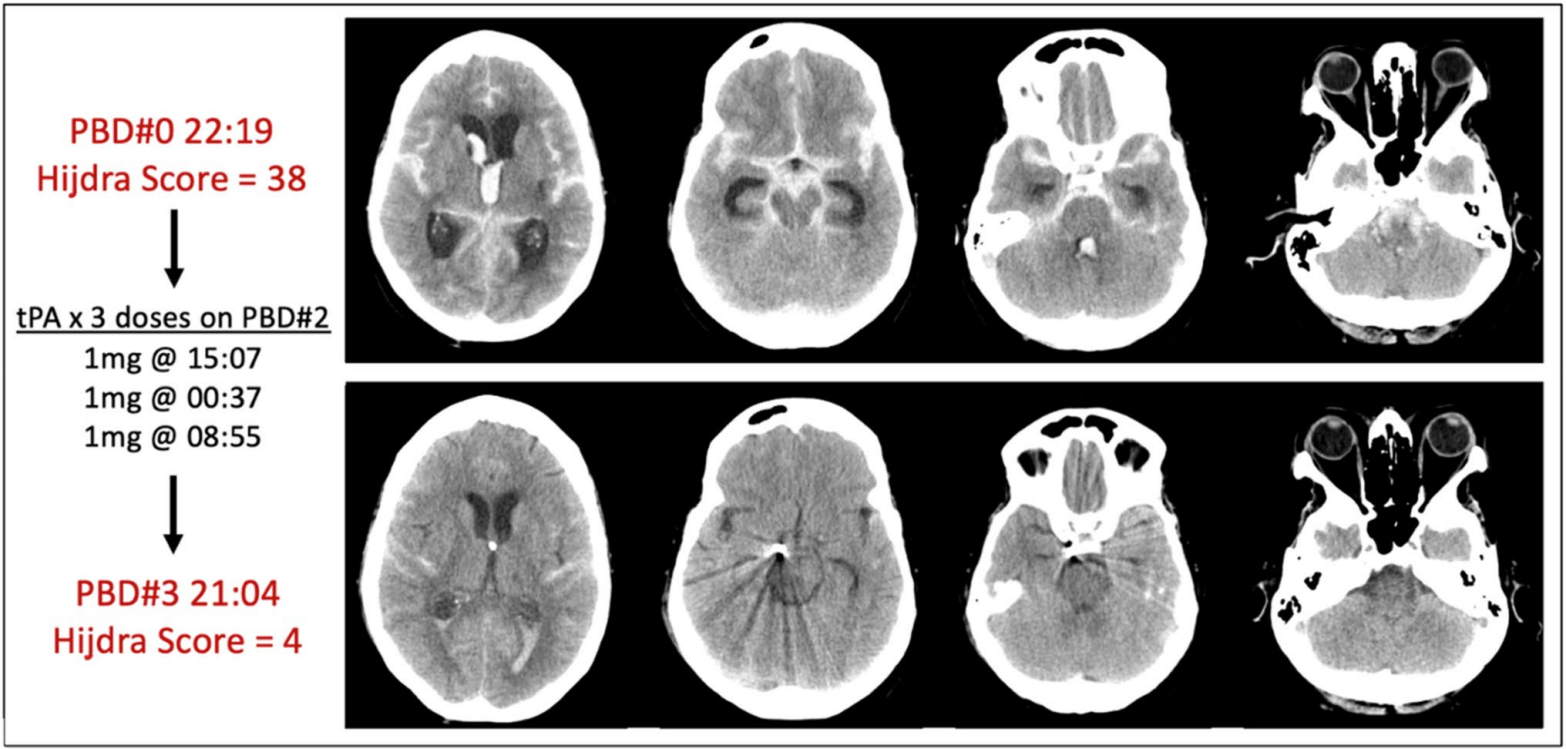
Patient with subarachnoid haemorrhage secondary to rupture of a right posterior communicating artery aneurysm with a modified Fisher score of 4. Following coil emobilisation, tissue plasminogen activator was administered over 3 doses, and this resulted in a reduction of Hijdra sum score from 38 to 4

The concept of intraventricular thrombolysis has also been tested in SAH. A randomised, open-label phase II study was performed to understand the effect of concomitant low-frequency head motion therapy and intraventricular fibrinolysis [38]. Patients were treated surgically or endovascularly and randomised to standard of care or additional intraventricular application of recombinant tissue-type plasminogen activator and low-frequency rotational therapy. There was a dual primary endpoint of functional outcome at both discharge and 3 months follow-up. While the intervention group showed a significantly faster clot clearance rate, there was no significant improvement in vasospasm, DCI, or the dual primary outcome. However, the sample size calculation was based on a risk reduction of 50% for symptomatic angiographic vasospasm and clinical features of DCI, rather than the dual primary outcome. Therefore, the study may have been under-powered to detect improvements in functional outcome. To address this limitation, a larger randomised clinical trial (RCT) of intraventricular thrombolysis following SAH is currently underway [39]. Figure 1 demonstrates the concept of intraventricular thrombolysis for SAH.

### Intracisternal Thrombolysis for SAH

The idea to accelerate clot resolution has also been tested in the intracisternal compartment. A cohort study recruited 436 patients, of whom 57 had an EVD catheter stereotactically implanted through the lateral ventricle, the foramen of Monro, and finally perforating the floor of the third ventricle [40]. CT confirmed correct catheter placement, and these patients subsequently received continuous cisternal lavage using a free-running electrolyte solution. Urokinase was added until macroscopic clearance of drainage fluid was observed, and nimodipine was added if there were clinical signs of delayed neurological deficit or sonographic vasospasm. 68% of these 57 patients had a favourable outcome at 6 months (mRS, ≤ 3), as compared to 222 patients who did not undergo catheter ventriculocisternostomy and lavage, where 53% had a favourable outcome.

A randomised trial tested the efficacy of intrathecal administration of urokinase into the cisterna magna to alleviate symptomatic vasospasm in SAH patients after coil embolisation [41]. A microcatheter was fluoroscopically introduced into the intrathecal space via lumbar puncture and advanced to the cisterna magna in the treatment group. The authors infused urokinase, clamped the catheter for one hour, and allowed drainage afterwards. The treatment was repeated after 12 h. There was a significant reduction in symptomatic vasospasm and shunt dependency in the treatment group. Furthermore, significantly more patients in the treatment group had favourable outcomes. Mortality rates did not differ between the two groups.

Another study recruited patients with aneurysmal SAH treated with clipping to evaluate the influence of cisternal irrigation therapy with tissue plasminogen activator on outcomes [42]. Lilliquist’s membrane was opened during surgery, and a cisternal drainage tube was placed in the basal cistern. Patients were randomly allocated to saline irrigation daily for 14 days, intermittent clot lysis every 8 h for 2 days, or continuous clot lysis via the cisternal catheter for 48 h. The clot clearance rate was defined by measuring the HUs postoperatively and on days 5 and 14 after SAH. They found a significantly higher clot clearance rate in the continuous and intermittent group than in the baseline group at day 5. There was no significant difference between the groups regarding cerebral infarction or white blood cell counts in the CSF. The intermittent group showed significantly better mRS outcomes at 3 months than the other two groups.

### Meta-analysis

A meta-analysis reviewed 21 studies administering thrombolytics (tissue plasminogen activator or urokinase) versus placebo or no treatment [43]. The methodology of different studies varied, including the type and dosage of thrombolytic agents. The authors performed separate analyses for intraventricular and intracisternal thrombolysis. The pooled analysis of patients who received intraventricular fibrinolysis showed no differences between the treatment and control groups regarding delayed ischemic neurologic deficit, functional outcome, shunt dependency, and bleeding complications. On the other hand, in the pooled analysis of patients treated with intracisternal fibrinolysis, the authors reported a significant reduction in delayed ischemic neurologic deficit, rate of poor functional outcomes, shunt dependency, and mortality. There was no increase in bleeding complications.

### Cisternal and Ventricular Compartmentalisation

There is a clear concept arising from the observational and interventional studies discussed above that blood clots within cisternal and ventricular compartments should be considered separately. In summary, cisternal (i.e. subarachnoid), but not ventricular clot clearance rate is associated with DCI and clinical outcome. Also, cisternal fibrinolysis may have a more pronounced effect on outcome compared to intraventricular thrombolysis. Hence, the compartmental localisation of the clot may be crucial in interpreting outcomes and planning treatments targeting clot clearance. For instance, it is now important to determine whether the priority should be to focus on rapid clearance of the cisternal clot.

## Lumbar Drains

**Fig. 2:**
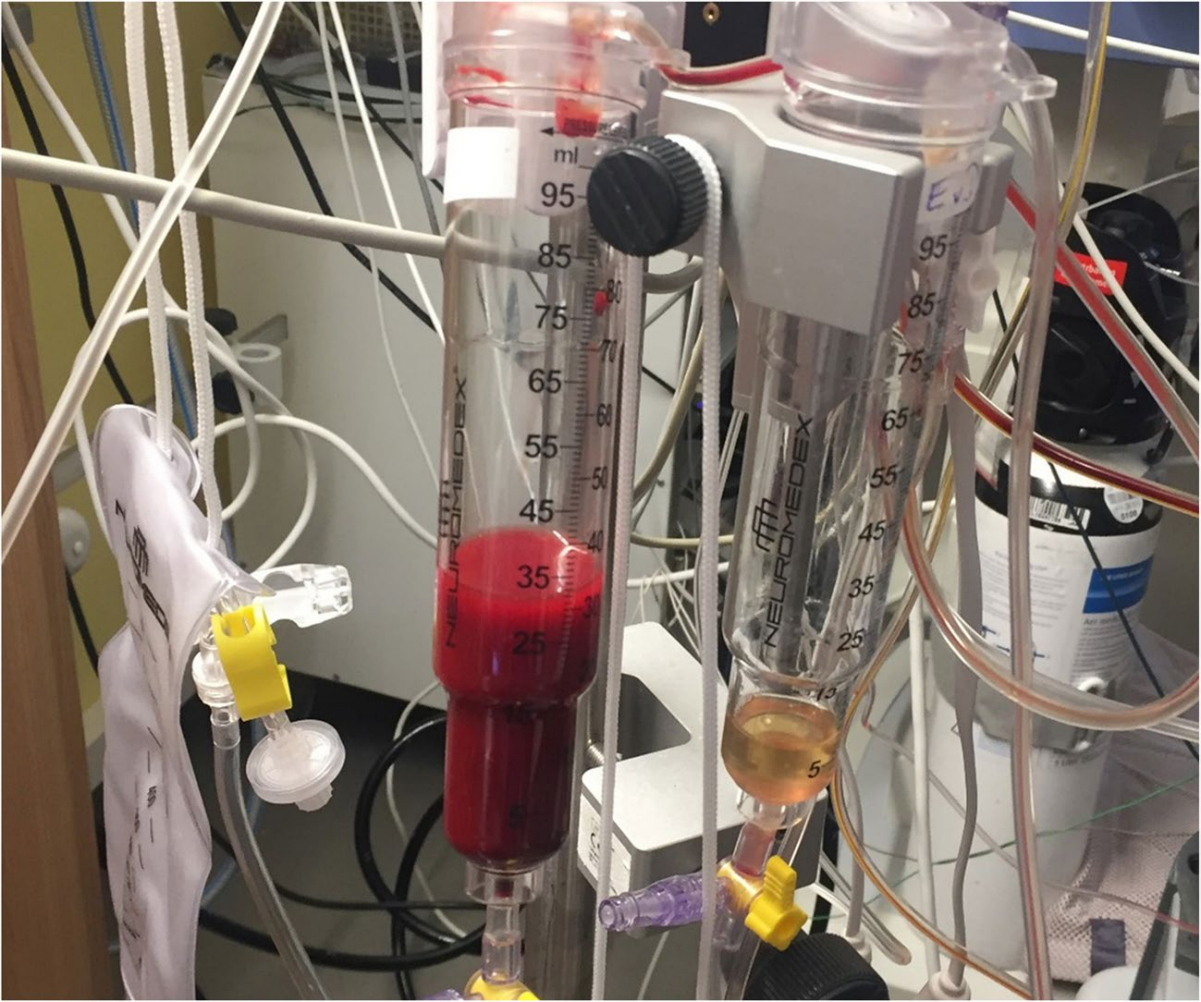
Drip chambers from a patient with aneurysmal subarachnoid haemorrhage, who had both an external ventricular drain (EVD) and lumbar drain in situ. Left side drip chamber from lumbar drain, right side drip chamber from EVD

Lumbar drains have long been used to drain CSF in SAH patients to varying degrees in units across the world. The aim is to treat hydrocephalus as well as drain blood-contaminated CSF in the hope that this reduces the levels of toxic byproducts of erythrolysis in the brain, thereby improving outcomes (Fig. 2). There has been a great deal of literature debating these practices over the years. Klimo Jr. et al. (2004) and Kwon et al. (2008) noted a marked reduction of clinical cerebral vasospasm in patients in whom a lumbar drain had been placed after SAH regardless of whether an EVD was present [44, 45]. The most recent meta-analysis on this topic has found that lumbar drain insertion in patients with SAH is associated with lower risk of cerebral vasospasm, delayed cerebral infarction, and death [46]. This positive impact of lumbar drain insertion may be mediated by an increased removal of RBCs and their breakdown products. Figure 2 corroborates reports from the literature that CSF within lumbar drains initially appears densely haemorrhagic and then progressively clear, whilst CSF from EVDs is considerably less haemorrhagic [44], indicating limited ability of EVDs compared to lumbar drains of removing RBCs and their breakdown products. This has not been formally quantified in existing clinical studies, but evidence from models suggests that a lumbar drain could remove 35% of RBCs from the CSF within 24 h [47].

Considering these promising results, a RCT of lumbar drains (LUMAS) was conducted. The trial protocol consisted of volume-driven CSF drainage using lumbar drains aiming for removal of 5 to 10 mL CSF per hour until the CSF was visibly clear. The LUMAS study had shown that lumbar drainage—thereby removing RBCs and their breakdown products—reduced the incidence of DCI from 35 to 21% and improved outcome at discharge, but had no effect on long-term outcomes [48]. However, they recruited less severely affected patients with lower risk of adverse outcomes and the study may thus have been underpowered to detect a significant effect. A non-randomised study conducted soon after the trial finished found that patients with a higher modified Fisher scale had significantly lower risk of cerebral vasospasm, delayed cerebral infarction, and hydrocephalus following insertion of a lumbar drain; but no difference was seen in patients with a modified Fisher scale of 0–2 [49]. Therefore, a further RCT was undertaken that included patients of all severities of SAH (EARLYDRAIN) and inserted lumbar drains irrespective of whether patients had an EVD already sited [50]. This showed that lumbar drainage (versus no drainage) was associated with an absolute risk reduction of secondary infarction and improved neurological outcomes both in the short and the long term. It is anticipated that these results will lead to a significant shift in clinical practice towards using lumbar drains.

## Neurapheresis

**Fig. 3:**
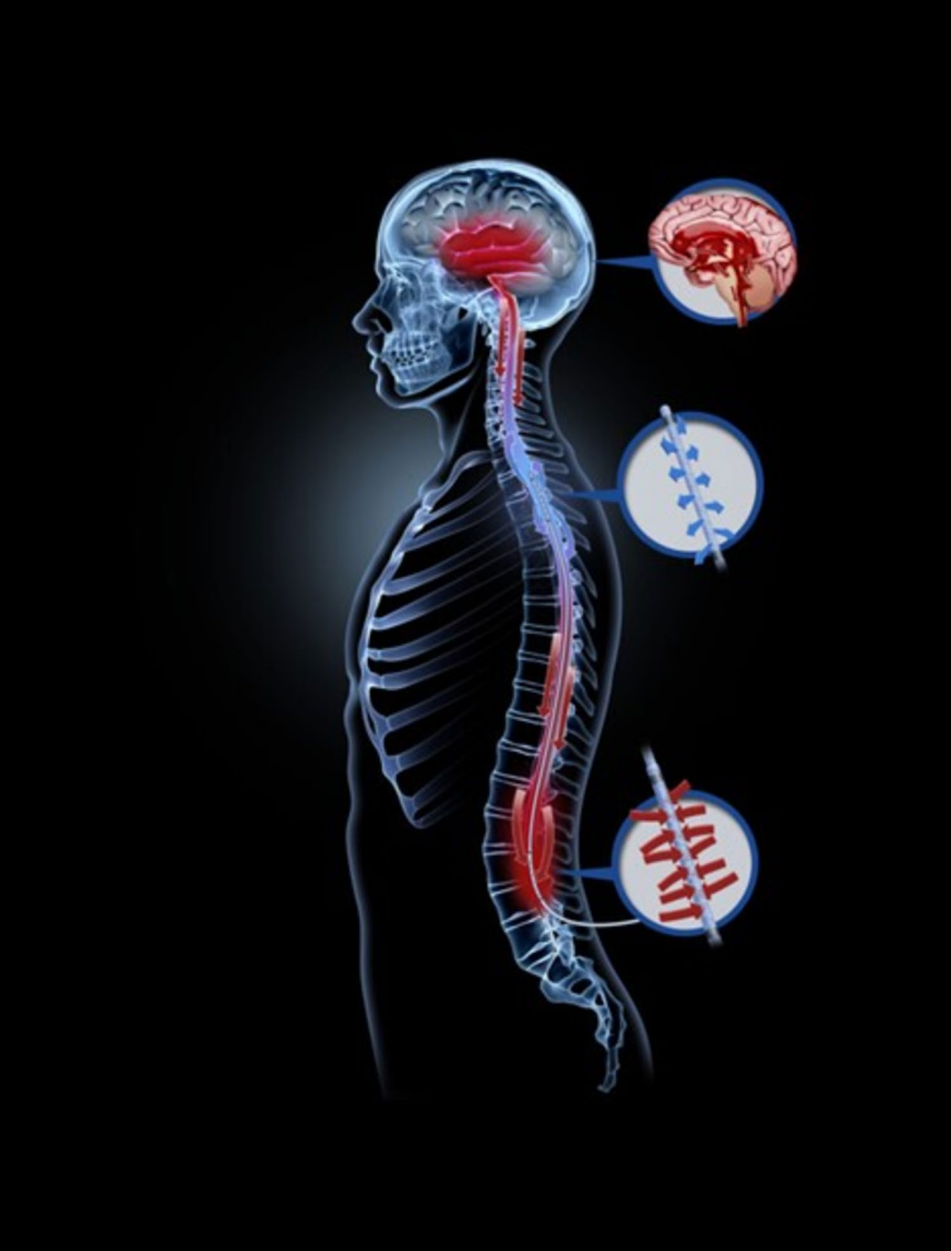
Schematic of the neurapheresis system

Neurapheresis therapy, a CSF filtration system, builds on the above findings. The technology involves aspiration of the blood-contaminated CSF from the lumbar cistern and then returning the filtered CSF to the thoracic subarachnoid space (Fig. 3), thereby removing RBCs and their cytotoxic products from the CSF. Neurapheresis overcomes the limitations of relying on natural production and circulation of CSF to remove RBCs, with fluid dynamic studies showing that a neurapheresis system at a maximum flow of 2.0 ml/min doubles the average steady streaming CSF velocity in comparison to lumbar drainage [51], and this could increase RBC clearance by 50% [47]. The PILLAR study is a notable first-in-human trial that evaluated the safety and feasibility of the neurapheresis system [52]. Results showed a significant reduction in CSF RBC counts and total protein after the filtration process. Moreover, CT scans demonstrated a decrease in cisternal blood. However, in the 3 patients with > 22 h of filtration, complete RBC clearance was not achieved (mean RBC reduction: 79.7%). To that end, the PILLAR-XT study (NCT03607825) will use longer pump times and will further characterise the treatment curve of neurapheresis therapy. It is also unclear the minimum size of substances that would be filtered out by the neurapheresis system. It may be that the neurapheresis system is able to remove RBCs, but breakdown products such as haemoglobin and haem remain within the CSF.

## EVD Wash Out

**Fig. 4:**
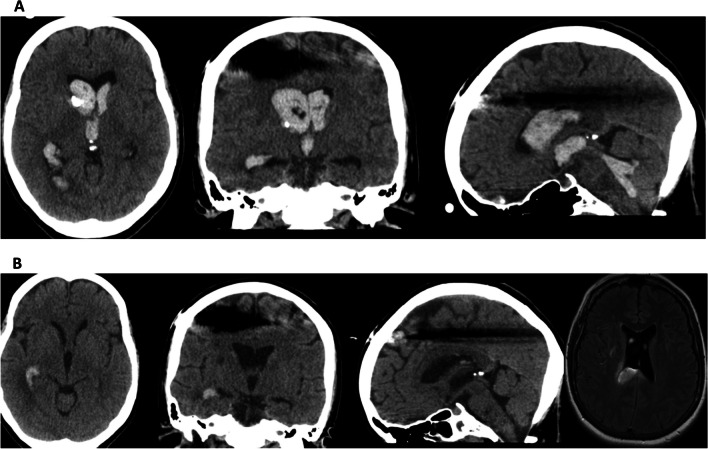
**A** Patient with intracranial haemorrhage and intraventricular haemorrhage before active cerebrospinal fluid exchange. The removal of blood was achieved in 47 h with a total of 2.4 mg tissue plasminogen activator in irrigation fluid. **B** MRI shows oedema only in part of brain parenchyma where removal of blood was not sufficient

**Fig. 5:**
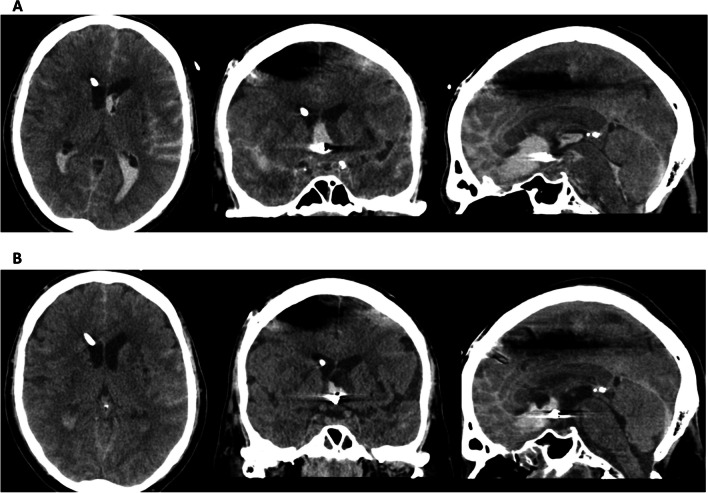
**A** Patient with high grade subarachnoid haemorrhage from an anterior communicating aneurysm. They underwent CSF exchange for 36 h with 2.2 mg tissue plasminogen activator. **B** Significant removal of blood is seen after CSF exchange. Despite the high chance of shunt dependency, the patient did not become shunt dependent in long-term follow-up

An alternative method for clearing CSF of toxic byproducts of erythrolysis following SAH involves employing a double lumen EVD in combination with CSF exchange (Fig. 4) [53]. The CSF exchange procedure can be conducted at a rate of up to 180 ml/h. This technique enables both active irrigation and passive drainage, providing simultaneous control over intracranial pressure. The most recently published trial on this technique was terminated early, however, due to a significantly increased risk of severe adverse events associated with intraventricular lavage at interim analysis [54]. It is important to note that approximately half of the patients recruited for this trial did not have SAH. A more focussed non-randomised study on SAH patients suggested that the use of the double lumen EVD was safe, and could potentially reduce shunt dependency after SAH (Fig. 5) [55]. Ongoing randomized trials, namely the Active Removal of Haemorrhagic Stroke (ARCH) and Vasospasm and Shunt Dependency (VASH), are continuing to explore the effects of rapidly removing blood from the CSF and its consequential impact in patients following SAH or ICH [56, 57]. Preliminary results seem to underscore the importance of the user's proficiency in operating the device.

## Haptoglobin

A recent review of the literature detailed the evidence supporting neurotoxicity of extracellular free haemoglobin released from RBCs when they lyse in the CSF [58]. Targeting of haemoglobin toxicity and scavenging is, therefore, a rational therapeutic strategy. Haptoglobin is an endogenous haemoglobin scavenger plasma protein in vertebrates. It irreversibly binds cell-free unbound haemoglobin in the plasma, forming large haptoglobin:haemoglobin complexes, which do not cross intercellular tight junctions or enter subendothelial spaces [14]. However, endogenous quantities of haptoglobin cannot match the amount of cell-free unbound haemoglobin that is released into the subarachnoid space following erythrolysis of RBCs in the CSF [59].

**Fig. 6:**
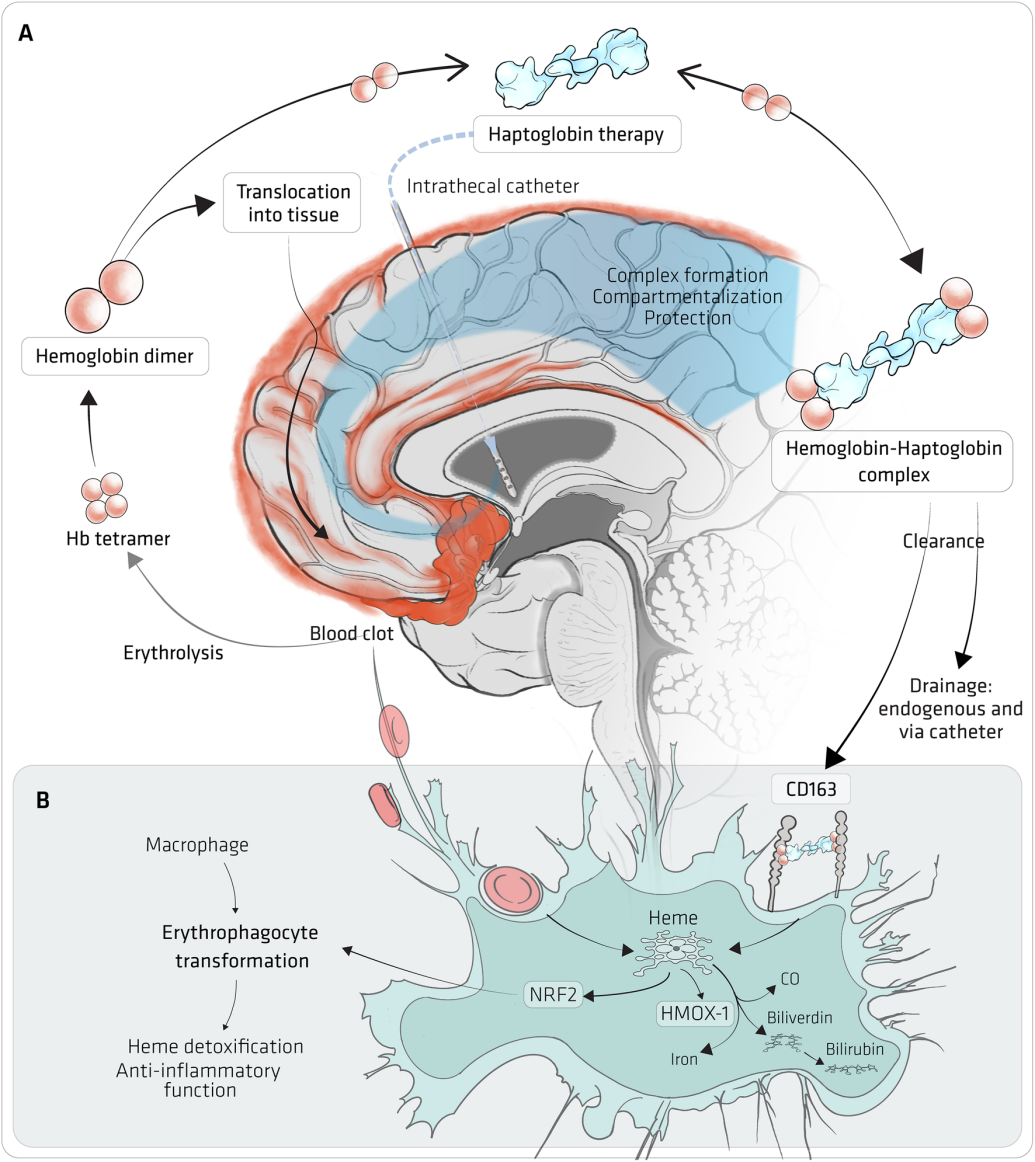
Haptoglobin treatment. **A** Erythrolysis releases haemoglobin tetramers, which dissociate into dimers in cerebrospinal fluid (CSF). Small Hb dimers penetrate the brain parenchyma and NO-sensitive arterial compartments to cause secondary brain injury. Therapeutic haptoglobin administered via an intrathecal catheter distributes throughout the CSF compartment and binds free haemoglobin. The large haemoglobin-haptoglobin complex remains confined outside the parenchyma and vulnerable arterial compartments, thereby protecting from haemoglobin-induced damage. The haemoglobin-haptoglobin complex is cleared by physiological drainage pathways and drained through intraventricular and/or lumbar catheters. **B** The role of macrophages in erythrophagocytosis and haemoglobin-haptoglobin complex clearance. Following degradation, haem is metabolised to bilirubin, carbon monoxide, and iron through haem-mediated induction of HMOX-1 (heme-oxygenase 1). Haem-induced activation of NRF2 (nuclear factor erythroid 2-related factor 2) signalling induces an anti-inflammatory macrophage phenotype (i.e. erythrophagocytosis). Taken from Galea et al. [65]

Nonaka et al. performed a small preliminary study of intrathecal administration of haptoglobin as a treatment for vasospasm in 27 patients with SAH in Japan, and improvements in vasospasm in some patients suggested some therapeutic benefits [60]. However, further exploration of intrathecal haptoglobin treatment for SAH by this group of researchers was abandoned for reasons that are unclear, and the exact composition of the intrathecal haptoglobin used is unknown. More recent experimental animal studies have demonstrated that intrathecal haptoglobin administration prevents small and large vessel spasm, neuronal toxicity and clinical deficits induced by exposure to extracellular haemoglobin [14, 61]. Furthermore, an in vitro study showed that haptoglobin can prevent haemoglobin neurotoxicity at sub-stoichiometric concentrations, enough to reduce the amount of free haemoglobin below a threshold concentration to prevent toxicity [62], in keeping with a previous observational study [63]. An ongoing prospective multicentre observational trial aims to validate this CSF-Hb threshold concentration in a large cohort of aSAH patients [64]. A recent consensus study has demonstrated that there is current widespread support to trial the delivery of exogenous haptoglobin directly into the CSF to neutralise excess total cell-free haemoglobin and determine if improvements in long-term clinical outcomes occur (Fig. 6) [65].

## Haemopexin

Unscavengeable haemoglobin eventually degrades into haem [13, 66]. Because of haem’s redox activity and lipophilicity, it can disrupt membrane homeostasis, causing cellular dysfunction and death [67]. Haem can be sequestered with very high affinity by haemopexin [68]. In a study involving 30 SAH patients, free haem was still detectable in the CSF after SAH, suggesting saturation of the haemopexin-CD91 system following SAH [69]. Similar to haptoglobin, intrathecal administration of haemopexin or selective agonists of haemopexin expression are possible therapeutic strategies to neutralise haem toxicity after brain haemorrhage.

A study where haem was both injected by itself and co-injected with haemopexin at a 1:1 stoichiometry into the brain of mice found that the presence of haemopexin prevented the haem-induced disruption of the blood–brain barrier, as quantified by dextran leakage, and attenuated haem-induced gene expression indicative of reactive astrogliosis [70]. Another study used a mouse model of ICH to show that increasing brain haemopexin levels using recombinant adeno-associated viral vectors significantly reduced tissue injury, astrogliosis and lipid peroxidation, and significantly increased microgliosis [71].

## Iron Chelation

Haem releases iron over time in SAH. While traditionally apoptosis was seen as the primary form of regulated cell death, recent research has shown that there are other non-apoptotic cell death pathways. A study by Dixon et al. identified a novel iron-dependent form of non-apoptotic cell death [72]. Minocycline and deferiprone are iron chelators that have been shown to reduce neuro-toxicity in vitro and in animal models of SAH [73–75]. Small RCTs in humans have been launched to determine their effectiveness after SAH.

## Transcriptional Upregulation of Anti-oxidative Responses

A peroxisome proliferator-activated receptor-γ (PPARγ) agonist, rosiglitazone, was initially shown in a mouse model of intracerebral haemorrhage to promote haematoma resolution, diminish neuronal damage, and aid recovery [76]. Rosiglitazone has since been used in a rat model of SAH, and it was found to reduce neuronal degeneration and improve outcomes [77]. PPARγ is a transcriptional activator of the gene that encodes nuclear factor-erythroid 2 p45-related factor 2 (NRF2). Genetic variation in NRF2 has been shown to be associated with outcome after SAH in humans [78] and NRF2 activation by sulforaphane, in animal models, reduced cerebral vasospasm, brain oedema, BBB leakage, cortical apoptosis, and motor deficits [79, 80]. A phase II clinical trial of sulforaphane (complexed with cyclodextrin) in SAH patients has been completed, and results are being prepared for publication [81].

## Conclusion

This review highlights the importance of a multifaceted approach in understanding and managing SAH, focusing on the initial blood load, clot clearance, and the management of RBC degradation by-products. Studies have indicated that the volume, density, and location of the haematoma are crucial factors in determining patient outcomes. Advancements in imaging techniques have improved the accuracy of prognosis predictions. Moreover, the kinetics of clot clearance have been identified as significant predictors of SAH-SBI and overall patient prognosis. The compartmental localisation of clots is crucial, with treatments like intracisternal thrombolysis showing promise in improving outcomes. The use of lumbar drains and innovative techniques like neurapheresis therapy also offer new avenues for reducing the burden of toxic by-products of erythrolysis, potentially improving outcomes. Furthermore, the potential of pharmacological agents, such as haptoglobin and haemopexin, in scavenging the neurotoxic components released from lysed RBCs, opens new doors for therapeutic interventions. The insights gained from current research pave the way for improved clinical outcomes and a better quality of life for SAH patients.

## Data Availability

Not applicable.

## References

[1] van Gijn J, Kerr RS, Rinkel GJ. Subarachnoid haemorrhage. Lancet (London, England). 2007;369:306–18. 10.1016/S0140-6736(07)60153-6. 17258671 10.1016/S0140-6736(07)60153-6

[2] Macdonald RL, Schweizer TA. Spontaneous subarachnoid haemorrhage. Lancet. 2017;389:655–66. 10.1016/S0140-6736(16)30668-7. 27637674 10.1016/S0140-6736(16)30668-7

[3] Etminan N, Chang HS, Hackenberg K, De Rooij NK, Vergouwen MDI, Rinkel GJE, Algra A. Worldwide incidence of aneurysmal subarachnoid hemorrhage according to region, time period, blood pressure, and smoking prevalence in the population: a systematic review and meta-analysis. JAMA Neurol. 2019;76:588–97. 10.1001/JAMANEUROL.2019.0006. 30659573 10.1001/jamaneurol.2019.0006 PMC6515606

[4] Taylor TN, Davis PH, Torner JC, Holmes J, Meyer JW, Jacobson MF. Lifetime cost of stroke in the United States. Stroke. 1996;27:1459–66. 10.1161/01.STR.27.9.1459. 8784113 10.1161/01.str.27.9.1459

[5] Connolly ES, Rabinstein AA, Carhuapoma JR, Derdeyn CP, Dion J, Higashida RT, Hoh BL, Kirkness CJ, Naidech AM, Ogilvy CS, et al. Guidelines for the management of aneurysmal subarachnoid hemorrhage: a guideline for healthcare professionals from the American Heart Association/American Stroke Association. Stroke. 2012;43:1711–37. 10.1161/STR.0B013E3182587839. 22556195 10.1161/STR.0b013e3182587839

[6] Rincon F, Rossenwasser RH, Dumont A. The epidemiology of admissions of nontraumatic subarachnoid hemorrhage in the United States. Neurosurgery. 2013;73:217–22. 10.1227/01.NEU.0000430290.93304.33. 23615089 10.1227/01.neu.0000430290.93304.33

[7] Koivisto T, Vanninen R, Hurskainen H, Saari T, Hernesniemi J, Vapalahti M. Outcomes of early endovascular versus surgical treatment of ruptured cerebral aneurysms. Stroke. 2000;31:2369–77. 10.1161/01.STR.31.10.2369. 11022066 10.1161/01.str.31.10.2369

[8] Pace A, Mitchell S, Casselden E, Zolnourian A, Glazier J, Foulkes L, Bulters D, Galea I. A subarachnoid haemorrhage-specific outcome tool. Brain. 2018;141:1111–21. 10.1093/BRAIN/AWY003. 29401245 10.1093/brain/awy003

[9] Wallmark S, Ronne-Engström E, Lundström E. Predicting return to work after subarachnoid hemorrhage using the Montreal Cognitive Assessment (MoCA). Acta Neurochir (Wien). 2016;158:233–9. 10.1007/S00701-015-2665-4. 26676517 10.1007/s00701-015-2665-4

[10] Hoh BL, Ko NU, Amin-Hanjani S, Hsiang-Yi Chou S, Cruz-Flores S, Dangayach NS, Derdeyn CP, Du R, Hänggi D, Hetts SW, et al. 2023 Guideline for the management of patients with aneurysmal subarachnoid hemorrhage: a guideline from the American Heart Association/American Stroke Association. Stroke. 2023;54:E314–70. 10.1161/STR.0000000000000436. 37212182 10.1161/STR.0000000000000436

[11] Theurl I, Hilgendorf I, Nairz M, Tymoszuk P, Haschka D, Asshoff M, He S, Gerhardt LMS, Holderried TAW, Seifert M, et al. On-demand erythrocyte disposal and iron recycling requires transient macrophages in the liver. Nat Med. 2016;22:945–51. 10.1038/NM.4146. 27428900 10.1038/nm.4146 PMC4957133

[12] Wan H, Brathwaite S, Ai J, Hynynen K, Macdonald RL. Role of perivascular and meningeal macrophages in outcome following experimental subarachnoid hemorrhage. J Cereb Blood Flow Metab. 2021;41:1842–57. 10.1177/0271678X20980296/ASSET/IMAGES/LARGE/10.1177_0271678X20980296-FIG7.JPEG. 33444089 10.1177/0271678X20980296 PMC8327101

[13] Dean L. Chapter 1 blood and the cells it contains [Internet]. Bethesda (MD): National Center for Biotechnology Information (US). 2005. Available from: https://www.ncbi.nlm.nih.gov/books/NBK2263/.

[14] Hugelshofer M, Buzzi RM, Schaer CA, Richter H, Akeret K, Anagnostakou V, Mahmoudi L, Vaccani R, Vallelian F, Deuel JW, et al. Haptoglobin administration into the subarachnoid space prevents hemoglobin-induced cerebral vasospasm. J Clin Invest. 2019;129:5219–35. 10.1172/JCI130630. 31454333 10.1172/JCI130630 PMC6877320

[15] Galea I, Durnford A, Glazier J, Mitchell S, Kohli S, Foulkes L, Norman J, Darekar A, Love S, Bulters DO, et al. Iron deposition in the brain after aneurysmal subarachnoid hemorrhage. Stroke. 2022;53:1633–42. 10.1161/STROKEAHA.121.036645. 35196874 10.1161/STROKEAHA.121.036645

[16] Woo PYM, Tse TPK, Chan RSK, Leung LNY, Liu SKK, Leung AYT, Wong HT, Chan KY. Computed tomography interobserver agreement in the assessment of aneurysmal subarachnoid hemorrhage and predictors for clinical outcome. J Neurointerv Surg. 2017;9:1118–24. 10.1136/NEURINTSURG-2016-012576. 29030464 10.1136/neurintsurg-2016-012576

[17] Jeong HG, Bang JS, Kim BJ, Bae HJ, Han MK. Hematoma Hounsfield units and expansion of intracerebral hemorrhage: a potential marker of hemostatic clot contraction. Int J Stroke. 2021;16:163–71. 10.1177/1747493019895703. 31992155 10.1177/1747493019895703

[18] Ishihara H, Oka F, Kawano R, Shinoyama M, Nishimoto T, Kudomi S, Suzuki M. Hounsfield unit value of interpeduncular cistern hematomas can predict symptomatic vasospasm. Stroke. 2020;51:143–8. 10.1161/STROKEAHA.119.026962. 31694506 10.1161/STROKEAHA.119.026962

[19] Friedman JA, Goerss SJ, Meyer FB, Piepgras DG, Pichelmann MA, McIver JI, Toussaint LG, McClelland RL, Nichols DA, Atkinson JLD, et al. Volumetric quantification of fisher grade 3 aneurysmal subarachnoid hemorrhage: a novel approach to predict symptomatic vasospasm on admission computerized tomography scans. J Neurosurg. 2002;97:401–7. 10.3171/JNS.2002.97.2.0401. 12186469 10.3171/jns.2002.97.2.0401

[20] Boers AM, Zijlstra IA, Gathier CS, Van Den Berg R, Slump CH, Marquering HA, Majoie CB. Automatic quantification of subarachnoid hemorrhage on noncontrast CT. AJNR Am J Neuroradiol. 2014;35:2279. 10.3174/AJNR.A4042. 25104292 10.3174/ajnr.A4042 PMC7965299

[21] Van Der Steen WE, Marquering HA, Ramos LA, Van Den Berg R, Coert BA, Boers AMM, Vergouwen MDI, Rinkel GJE, Velthuis BK, Roos YBWEM, et al. Prediction of outcome using quantified blood volume in aneurysmal SAH. Am J Neuroradiol. 2020;41:1015–21. 10.3174/AJNR.A6575. 32409315 10.3174/ajnr.A6575 PMC7342737

[22] Rosen DS, Macdonald RL, Huo D, Goldenberg FD, Novakovic RL, Frank JI, Rosengart AJ. Intraventricular hemorrhage from ruptured aneurysm: clinical characteristics, complications, and outcomes in a large, prospective, multicenter study population. J Neurosurg. 2007;107:261–5. 10.3171/JNS-07/08/0261. 17695378 10.3171/JNS-07/08/0261

[23] Kramer AH, Mikolaenko I, Deis N, Dumont AS, Kassell NF, Bleck TP, Nathan BA. Intraventricular hemorrhage volume predicts poor outcomes but not delayed ischemic neurological deficits among patients with ruptured cerebral aneurysms. Neurosurgery. 2010;67:1044–52. 10.1227/NEU.0B013E3181ED1379. 20881568 10.1227/NEU.0b013e3181ed1379

[24] Ko SB, Choi HA, Carpenter AM, Helbok R, Schmidt JM, Badjatia N, Claassen J, Connolly ES, Mayer SA, Lee K. Quantitative analysis of hemorrhage volume for predicting delayed cerebral ischemia after subarachnoid hemorrhage. Stroke. 2011;42:669–74. 10.1161/STROKEAHA.110.600775. 21257823 10.1161/STROKEAHA.110.600775

[25] Van Der Steen WE, Zijlstra IA, Verbaan D, Boers AMM, Gathier CS, Van Den Berg R, Rinkel GJE, Coert BA, Roos YBWEM, Majoie CBLM, et al. Association of quantified location-specific blood volumes with delayed cerebral ischemia after aneurysmal subarachnoid hemorrhage. AJNR Am J Neuroradiol. 2018;39:1059. 10.3174/AJNR.A5626. 29650786 10.3174/ajnr.A5626 PMC7410623

[26] Horst V, Kola V, Lemale CL, Major S, Winkler MKL, Hecht N, Santos E, Platz J, Sakowitz OW, Vatter H, et al. Spreading depolarization and angiographic spasm are separate mediators of delayed infarcts. Brain Commun. 2023;5(2):fcad080. 10.1093/braincomms/fcad080. 10.1093/braincomms/fcad080 PMC10082345 37038498

[27] Luo C, Yao X, Li J, He B, Liu Q, Ren H, Liang F, Li M, Lin H, Peng J, et al. Paravascular pathways contribute to vasculitis and neuroinflammation after subarachnoid hemorrhage independently of glymphatic control. Cell Death Dis. 2016;73(7):e2160–e2160. 10.1038/cddis.2016.63. 10.1038/cddis.2016.63 PMC4823962 27031957

[28] Lindner A, Berek K, Rass V, Di Pauli F, Kofler M, Zinganell A, Putnina L, Kindl P, Schiefecker AJ, Pfausler B, et al. Lower initial red blood cell count in cerebrospinal fluid predicts good functional outcome in patients with spontaneous subarachnoid haemorrhage. Eur J Neurol. 2023;30:2315–23. 10.1111/ENE.15845. 37161833 10.1111/ene.15845

[29] Zinganell A, Bsteh G, Di Pauli F, Rass V, Helbok R, Walde J, Deisenhammer F, Hegen H. Longitudinal ventricular cerebrospinal fluid profile in patients with spontaneous subarachnoid hemorrhage. Front Neurol. 2022;26(13):861625. 10.3389/fneur.2022.861625. 10.3389/fneur.2022.861625 PMC9360751 35959383

[30] Lenski M, Biczok A, Huge V, Forbrig R, Briegel J, Tonn JC, Thon N. Role of Cerebrospinal fluid markers for predicting shunt-dependent hydrocephalus in patients with subarachnoid hemorrhage and external ventricular drain placement. World Neurosurg. 2019;121:e535–42. 10.1016/J.WNEU.2018.09.159. 30268545 10.1016/j.wneu.2018.09.159

[31] Naff NJ, Williams MA, Rigamonti D, Keyl PM, Hanley DF. Blood clot resolution in human cerebrospinal fluid: evidence of first-order kinetics. Neurosurgery. 2001;49:614–21. 10.1097/00006123-200109000-00015. 11523671 10.1097/00006123-200109000-00015

[32] Zeineddine HA, Divito A, McBride DW, Pandit P, Capone S, Dawes BH, Chen CJ, Grotta JC, Blackburn SL. Subarachnoid blood clearance and aneurysmal subarachnoid hemorrhage outcomes: a retrospective review. Neurocrit Care. 2023;39:172–9. 10.1007/S12028-023-01729-X. 37100974 10.1007/s12028-023-01729-x

[33] Ritzenthaler T, Gobert F, Bouchier B, Dailler F. Amount of blood during the subacute phase and clot clearance rate as prognostic factors for delayed cerebral ischemia after aneurysmal subarachnoid hemorrhage. J Clin Neurosci. 2021;87:74–9. 10.1016/J.JOCN.2021.02.007. 33863538 10.1016/j.jocn.2021.02.007

[34] Reilly C, Amidei C, Tolentino J, Jahromi BS, Macdonald RL. Clot volume and clearance rate as independent predictors of vasospasm after aneurysmal subarachnoid hemorrhage. J Neurosurg. 2004;101:255–61. 10.3171/JNS.2004.101.2.0255. 15309916 10.3171/jns.2004.101.2.0255

[35] Hanley DF, Lane K, McBee N, Ziai W, Tuhrim S, Lees KR, Dawson J, Gandhi D, Ullman N, Mould WA, et al. Thrombolytic removal of intraventricular haemorrhage in treatment of severe stroke: results of the randomised, multicentre, multiregion, placebo-controlled CLEAR III Trial. Lancet (London, England). 2017;389:603–11. 10.1016/S0140-6736(16)32410-2. 28081952 10.1016/S0140-6736(16)32410-2 PMC6108339

[36] Gusdon AM, Thompson CB, Quirk K, Mayasi YM, Avadhani R, Awad IA, Hanley DF, Ziai WC. CSF and serum inflammatory response and association with outcomes in spontaneous intracerebral hemorrhage with intraventricular extension: An analysis of the CLEAR-III trial. J Neuroinflammation. 2021;18(1):179. 10.1186/s12974-021-02224-w. 34419101 10.1186/s12974-021-02224-w PMC8380363

[37] Fam MD, Zeineddine HA, Eliyas JK, Stadnik A, Jesselson M, McBee N, Lane K, Cao Y, Wu M, Zhang L, et al. CSF inflammatory response after intraventricular hemorrhage. Neurology. 2017;89:1553. 10.1212/WNL.0000000000004493. 28887375 10.1212/WNL.0000000000004493 PMC5634667

[38] Etminan N, Beseoglu K, Eicker SO, Turowski B, Steiger HJ, Hänggi D. Prospective, randomized, open-label phase II trial on concomitant intraventricular fibrinolysis and low-frequency rotation after severe subarachnoid hemorrhage. Stroke. 2013;44:2162–8. 10.1161/STROKEAHA.113.001790. 23735957 10.1161/STROKEAHA.113.001790

[39] Gaberel T, Gakuba C, Fournel F, Le Blanc E, Gaillard C, Saint Paul LP, Chaillot F, Tanguy P, Parienti JJ, Emery E. FIVHeMA: intraventricular fibrinolysis versus external ventricular drainage alone in aneurysmal subarachnoid hemorrhage: a randomized controlled trial. Neurochirurgie. 2019;65:14–9. 10.1016/J.NEUCHI.2018.11.004. 30638547 10.1016/j.neuchi.2018.11.004

[40] Roelz R, Schaefer JH, Scheiwe C, Sajonz B, Csok I, Steiert C, Buttler J, Rohr E, Grauvogel J, Shah MJ, et al. Impact of stereotactic ventriculocisternostomy on delayed cerebral infarction and outcome after subarachnoid hemorrhage. Stroke. 2020;51:431–9. 10.1161/STROKEAHA.119.027424. 31795898 10.1161/STROKEAHA.119.027424

[41] Hamada JI, Kai Y, Morioka M, Yano S, Mizuno T, Hirano T, Kazekawa K, Ushio Y. Effect on cerebral vasospasm of coil embolization followed by microcatheter intrathecal urokinase infusion into the cisterna magna: a prospective randomized study. Stroke. 2003;34:2549–54. 10.1161/01.STR.0000094731.63690.FF. 14563967 10.1161/01.STR.0000094731.63690.FF

[42] Yamamoto T, Esaki T, Nakao Y, Mori K. Efficacy of Low-dose tissue-plasminogen activator intracisternal administration for the prevention of cerebral vasospasm after subarachnoid hemorrhage. World Neurosurg. 2010;73:675–82. 10.1016/J.WNEU.2010.04.002. 20934155 10.1016/j.wneu.2010.04.002

[43] Lu X, Ji C, Wu J, You W, Wang W, Wang Z, Chen G. Intrathecal fibrinolysis for aneurysmal subarachnoid hemorrhage: evidence from randomized controlled trials and cohort studies. Front Neurol. 2019;10:885. 10.3389/FNEUR.2019.00885/FULL. 31481923 10.3389/fneur.2019.00885 PMC6709660

[44] Klimo P, Kestle JRW, Macdonald JD, Schmidt RH. Marked reduction of cerebral vasospasm with lumbar drainage of cerebrospinal fluid after subarachnoid hemorrhage. J Neurosurg. 2004;100:215–24. 10.3171/JNS.2004.100.2.0215. 15086227 10.3171/jns.2004.100.2.0215

[45] Kwon OY, Kim YJ, Kim YJ, Cho CS, Lee SK, Cho MK. The utility and benefits of external lumbar CSF drainage after endovascular coiling on aneurysmal subarachnoid hemorrhage. J Korean Neurosurg Soc. 2008;43:281. 10.3340/JKNS.2008.43.6.281. 19096633 10.3340/jkns.2008.43.6.281 PMC2588254

[46] Hulou MM, Essibayi MA, Benet A, Lawton MT. Lumbar drainage after aneurysmal subarachnoid hemorrhage: a systematic review and meta-analysis. World Neurosurg. 2022;166:261-267.e9. 10.1016/J.WNEU.2022.07.061. 35868504 10.1016/j.wneu.2022.07.061

[47] Khani M, Sass LR, Sharp MK, McCabe AR, Zitella Verbick LM, Lad SP, Martin BA. In vitro and numerical simulation of blood removal from cerebrospinal fluid: comparison of lumbar drain to neurapheresis therapy. Fluids Barriers CNS. 2020;17:1–17. 10.1186/S12987-020-00185-5/FIGURES/7. 32178689 10.1186/s12987-020-00185-5 PMC7077023

[48] Al-Tamimi YZ, Bhargava D, Feltbower RG, Hall G, Goddard AJP, Quinn AC, Ross SA. Lumbar drainage of cerebrospinal fluid after aneurysmal subarachnoid hemorrhage: a prospective, randomized, controlled trial (LUMAS). Stroke. 2012;43:677–82. 10.1161/STROKEAHA.111.625731. 22282887 10.1161/STROKEAHA.111.625731

[49] Fang Y, Shao Y, Lu J, Dong X, Zhao X, Zhang J, Chen S. The effectiveness of lumbar cerebrospinal fluid drainage in aneurysmal subarachnoid hemorrhage with different bleeding amounts. Neurosurg Rev. 2020;43:739–47. 10.1007/S10143-019-01116-1/FIGURES/3. 31161445 10.1007/s10143-019-01116-1

[50] Wolf S, Mielke D, Barner C, Malinova V, Kerz T, Wostrack M, Czorlich P, Salih F, Engel DC, Ehlert A, et al. Effectiveness of lumbar cerebrospinal fluid drain among patients with aneurysmal subarachnoid hemorrhage: a randomized clinical trial. JAMA Neurol. 2023. 10.1001/JAMANEUROL.2023.1792. 37330974 10.1001/jamaneurol.2023.1792 PMC10277935

[51] Khani M, Sass LR, McCabe AR, Zitella Verbick LM, Lad SP, Sharp MK, Martin BA. Impact of neurapheresis system on intrathecal cerebrospinal fluid dynamics: A computational fluid dynamics study. J Biomech Eng. 2020;142(2):0210061–9. 10.1115/1.4044308. 31343659 10.1115/1.4044308 PMC7104775

[52] Blackburn SL, Grande AW, Swisher CB, Hauck EF, Jagadeesan B, Provencio JJ. Prospective trial of cerebrospinal fluid filtration after aneurysmal subarachnoid hemorrhage via lumbar catheter (PILLAR). Stroke. 2019;50:2558–61. 10.1161/STROKEAHA.119.025399. 31345133 10.1161/STROKEAHA.119.025399 PMC6710124

[53] Garavaglia J, Hardigan T, Turner R, Monachello G, Khan MB, Hodge JO, Brandmeir NJ. Continuous intrathecal medication delivery with the IRRAflow catheter: Pearls and early experience. Oper Neurosurg (Hagerstown, Md). 2024;26(3):293–300. 10.1227/ons.0000000000000940. 10.1227/ons.0000000000000940 37819074

[54] Haldrup M, Rasmussen M, Mohamad N, Dyrskog S, Thorup L, Mikic N, Wismann J, Grønhøj M, Poulsen FR, Nazari M, et al. Intraventricular lavage vs external ventricular drainage for intraventricular hemorrhage: a randomized clinical trial. JAMA Netw open. 2023;6:e2335247. 10.1001/JAMANETWORKOPEN.2023.35247. 37815832 10.1001/jamanetworkopen.2023.35247 PMC10565600

[55] Jahromi BR, Brandmeir N, Göhre F, Niemelä M, Tanskanen P, Siironen J. Active CSF exchange and removal of blood after aneurysmatic SAH reduces shunt dependency. Brain and Spine. 2023;3:101805. 10.1016/J.BAS.2023.101805.

[56] Martin, W. IRRAS accounces announces first patient enrolled in ARCH Study, a comparative clinical trial designed to confirm IRRAflow’s ability to improve clearance of clot after IVH – IRRAS. 2021. Available online: https://news.cision.com/irras/r/irras-accounces-announces-first-patient-enrolled-in-arch-study%2D%2Dacomparative-clinical-trial-designe,c3442055. Accessed 20 Dec 2023.

[57] IRRAS. IRRAS Annual Report 2022. 2022. Available online: https://mb.cision.com/Main/16550/3759350/2016290.pdf.

[58] Bulters D, Gaastra B, Zolnourian A, Alexander S, Ren D, Blackburn SL, Borsody M, Doré S, Galea J, Iihara K, et al. Haemoglobin scavenging in intracranial bleeding: biology and clinical implications. Nat Rev Neurol. 2018;14:416–32. 10.1038/s41582-018-0020-0. 29925923 10.1038/s41582-018-0020-0

[59] Galea J, Cruickshank G, Teeling JL, Boche D, Garland P, Perry VH, Galea I. The intrathecal CD163-haptoglobin–hemoglobin scavenging system in subarachnoid hemorrhage. J Neurochem. 2012;121:785–92. 10.1111/J.1471-4159.2012.07716.X. 22380637 10.1111/j.1471-4159.2012.07716.x PMC3412209

[60] Nonaka T, Watanabe S, Chigasaki H, Miyaoka M, Ishii S. Etiology and treatment of vasospasm following subarachnoid hemorrhage. Neurol Med Chir (Tokyo). 1979;19:53–60. 10.2176/NMC.19.53. 84356 10.2176/nmc.19.53

[61] Garland P, Morton MJ, Haskins W, Zolnourian A, Durnford A, Gaastra B, Toombs J, Heslegrave AJ, More J, Okemefuna AI, et al. Haemoglobin causes neuronal damage in vivo which is preventable by haptoglobin. Brain Commun. 2020;2(1):fcz053. 10.1093/braincomms/fcz053. PMC7188517 32346673

[62] Warming H, Deinhardt K, Garland P, More J, Bulters D, Galea I, Vargas-Caballero M. Functional effects of haemoglobin can be rescued by haptoglobin in an in vitro model of subarachnoid haemorrhage. J Neurochem. 2023;167(1):90–103. 10.1111/jnc.15936. 37702203 10.1111/jnc.15936

[63] Akeret K, Buzzi RM, Schaer CA, Thomson BR, Vallelian F, Wang S, Willms J, Sebök M, Held U, Deuel JW, et al. Cerebrospinal fluid hemoglobin drives subarachnoid hemorrhage-related secondary brain injury. J Cereb Blood Flow Metab. 2021;41:3000–15. 10.1177/0271678X211020629. 34102922 10.1177/0271678X211020629 PMC8545037

[64] Akeret K, Buzzi RM, Saxenhofer M, Bieri K, Chiavi D, Thomson BR, Grüttner-Durmaz M, Schwendinger N, Humar R, Regli L, et al. The HeMoVal study protocol: a prospective international multicenter cohort study to validate cerebrospinal fluid hemoglobin as a monitoring biomarker for aneurysmal subarachnoid hemorrhage related secondary brain injury. BMC Neurol. 2022;22:1–13. 35850705 10.1186/s12883-022-02789-w PMC9290286

[65] Galea I, Bandyopadhyay S, Bulters D, Humar R, Hugelshofer M, Schaer DJ, Abdulazim A, Alalade AF, Alexander SA, Amaro S, et al. Haptoglobin treatment for aneurysmal subarachnoid hemorrhage: review and expert consensus on clinical translation. Stroke. 2023;54:1930–42. 10.1161/strokeaha.123.040205. 37232189 10.1161/STROKEAHA.123.040205 PMC10289236

[66] Joerk A, Ritter M, Langguth N, Seidel RA, Freitag D, Herrmann KH, Schaefgen A, Ritter M, Günther M, Sommer C, et al. Propentdyopents as heme degradation intermediates constrict mouse cerebral arterioles and are present in the cerebrospinal fluid of patients with subarachnoid hemorrhage. Circ Res. 2019;124:e101–14. 10.1161/CIRCRESAHA.118.314160. 30947629 10.1161/CIRCRESAHA.118.314160

[67] Chiabrando D, Vinchi F, Fiorito V, Mercurio S, Tolosano E. Heme in pathophysiology: a matter of scavenging, metabolism and trafficking across cell membranes. Front Pharmacol. 2014;5:1–24. 10.3389/FPHAR.2014.00061. 24782769 10.3389/fphar.2014.00061 PMC3986552

[68] Paoli M, Anderson BF, Baker HM, Morgan WT, Smith A, Baker EN. (1996) Crystal structure of hemopexin reveals a novel high-affinity heme site formed between two β-propeller domains. Nat Struct Biol. 1999;610(6):926–31. 10.1038/13294. 10.1038/13294 10504726

[69] Garland P, Durnford AJ, Okemefuna AI, Dunbar J, Nicoll JAR, Galea J, Boche D, Bulters DO, Galea I. Heme-hemopexin scavenging is active in the brain and associates with outcome after subarachnoid hemorrhage. Stroke. 2016;47:872–6. 10.1161/STROKEAHA.115.011956. 26768209 10.1161/STROKEAHA.115.011956

[70] Buzzi RM, Akeret K, Schwendinger N, Klohs J, Vallelian F, Hugelshofer M, Schaer DJ. Spatial transcriptome analysis defines heme as a hemopexin-targetable inflammatoxin in the brain. Free Radic Biol Med. 2022;179:277–87. 10.1016/J.FREERADBIOMED.2021.11.011. 34793930 10.1016/j.freeradbiomed.2021.11.011

[71] Leclerc JL, Santiago-Moreno J, Dang A, Lampert AS, Cruz PE, Rosario AM, Golde TE, Doré S. Increased brain hemopexin levels improve outcomes after intracerebral hemorrhage. J Cereb Blood Flow Metab. 2018;38:1032–46. 10.1177/0271678X16679170/ASSET/IMAGES/LARGE/10.1177_0271678X16679170-FIG6.JPEG. 27864463 10.1177/0271678X16679170 PMC5999006

[72] Dixon SJ, Lemberg KM, Lamprecht MR, Skouta R, Zaitsev EM, Gleason CE, Patel DN, Bauer AJ, Cantley AM, Yang WS, et al. Ferroptosis: an iron-dependent form of nonapoptotic cell death. Cell. 2012;149:1060–72. 10.1016/J.CELL.2012.03.042. 22632970 10.1016/j.cell.2012.03.042 PMC3367386

[73] Arthur AS, Fergus AH, Lanzino G, Mathys J, Kassell NF, Lee KS. Systemic administration of the iron chelator deferiprone attenuates subarachnoid hemorrhage-induced cerebral vasospasm in the rabbit. Neurosurgery. 1997;41:1385–92. 10.1097/00006123-199712000-00028. 9402590 10.1097/00006123-199712000-00028

[74] Liu H, Schwarting J, Terpolilli NA, Nehrkorn K, Plesnila N. Scavenging free iron reduces arteriolar microvasospasms after experimental subarachnoid hemorrhage. Stroke. 2021;52:4033–42. 10.1161/STROKEAHA.120.033472. 34749506 10.1161/STROKEAHA.120.033472

[75] Li J, Chen S, Fan J, Zhang G, Ren R. Minocycline attenuates experimental subarachnoid hemorrhage in rats. Open Life Sci. 2019;14:595. 10.1515/BIOL-2019-0067. 33817197 10.1515/biol-2019-0067 PMC7874754

[76] Zhao X, Sun G, Zhang J, Strong R, Song W, Gonzales N, Grotta JC, Aronowski J. Hematoma resolution as a target for intracerebral hemorrhage treatment: role for peroxisome proliferator-activated receptor gamma in microglia/macrophages. Ann Neurol. 2007;61:352–62. 10.1002/ANA.21097. 17457822 10.1002/ana.21097

[77] Lin BF, Kuo CY, Wen LL, Chen CM, Chang YF, Wong CS, Cherng CH, Chuang MY, Wu ZF. Rosiglitazone attenuates cerebral vasospasm and provides neuroprotection in an experimental rat model of subarachnoid hemorrhage. Neurocrit Care. 2014;21:316–31. 10.1007/S12028-014-0010-Z. 25022803 10.1007/s12028-014-0010-z

[78] Gaastra B, Duncan P, Bakker MK, Hostettler IC, Alg VS, Houlden H, Ruigrok YM, Galea I, Tapper W, Werring D, et al. Genetic variation in NFE2L2 is associated with outcome following aneurysmal subarachnoid haemorrhage. Eur J Neurol. 2023;30:116–24. 10.1111/ENE.15571. 36148820 10.1111/ene.15571 PMC10092511

[79] Zhao X, Wen L, Dong M, Lu X. Sulforaphane activates the cerebral vascular Nrf2-ARE pathway and suppresses inflammation to attenuate cerebral vasospasm in rat with subarachnoid hemorrhage. Brain Res. 2016;1653:1–7. 10.1016/J.BRAINRES.2016.09.035. 27693416 10.1016/j.brainres.2016.09.035

[80] Chen G, Fang Q, Zhang J, Zhou D, Wang Z. Role of the Nrf2-ARE pathway in early brain injury after experimental subarachnoid hemorrhage. J Neurosci Res. 2011;89:515–23. 10.1002/JNR.22577. 21259333 10.1002/jnr.22577

[81] Zolnourian AH, Franklin S, Galea I, Bulters DO. Study protocol for SFX-01 after subarachnoid haemorrhage (SAS): A multicentre randomised double-blinded, placebo controlled trial. BMJ Open. 2020;10(3):e028514. 10.1136/bmjopen-2018-028514. 32217557 10.1136/bmjopen-2018-028514 PMC7170552

